# A new tool for the chemical genetic investigation of the *Plasmodium falciparum* Pfnek-2 NIMA-related kinase

**DOI:** 10.1186/s12936-016-1580-3

**Published:** 2016-11-07

**Authors:** Deborah F. Mitcheson, Andrew R. Bottrill, Katherine Carr, Christopher R. Coxon, Celine Cano, Bernard T. Golding, Roger J. Griffin, Andrew M. Fry, Christian Doerig, Richard Bayliss, Andrew B. Tobin

**Affiliations:** 1 Department of Molecular and Cell Biology, University of Leicester, Lancaster Road, Leicester, LE1 9HN UK; 2 Protein and Nucleic Acid Chemistry Laboratory, University of Leicester, Hodgkin Building, Leicester, LE1 9HN UK; 3 School of Pharmacy and Biomolecular Sciences, Liverpool John Moores University, 2 Rodney Street, Liverpool, L3 5UX UK; 4 School of Chemistry, Bedson Building, Northern Institute for Cancer Research, Newcastle University, Newcastle upon Tyne, NE1 7RU UK; 5 Department of Microbiology, Building 76, Monash University, Wellington Road, Clayton, VIC 3800 Australia; 6 Astbury 6.108a, School of Molecular and Cellular Biology, University of Leeds, Leeds, LS2 9JT UK; 7 Centre for Translational Pharmacology, Institute of Molecular Cell and Systems Biology, University of Glasgow, Glasgow, G12 8QQ UK

**Keywords:** *Plasmodium falciparum*, NIMA-related protein kinase (Nek), Covalent modification, Chemical genetics, Mass spectrometry

## Abstract

**Background:**

Examining essential biochemical pathways in *Plasmodium falciparum* presents serious challenges, as standard molecular techniques such as siRNA cannot be employed in this organism, and generating gene knock-outs of essential proteins requires specialized conditional approaches. In the study of protein kinases, pharmacological inhibition presents a feasible alternative option. However, as in mammalian systems, inhibitors often lack the desired selectivity. Described here is a chemical genetic approach to selectively inhibit Pfnek-2 in *P. falciparum,* a member of the NIMA-related kinase family that is essential for completion of the sexual development of the parasite.

**Results:**

Introduction of a valine to cysteine mutation at position 24 in the glycine rich loop of Pfnek-2 does not affect kinase activity but confers sensitivity to the protein kinase inhibitor 4-(6-ethynyl-9H-purin-2-ylamino) benzene sulfonamide (NCL-00016066). Using a combination of in vitro kinase assays and mass spectrometry, (including phosphoproteomics) the study shows that this compound acts as an irreversible inhibitor to the mutant Pfnek2 likely through a covalent link with the introduced cysteine residue. In particular, this was shown by analysis of total protein mass using mass spectrometry which showed a shift in molecular weight of the mutant kinase in the presence of the inhibitor to be precisely equivalent to the molecular weight of NCL-00016066. A similar molecular weight shift was not observed in the wild type kinase. Importantly, this inhibitor has little activity towards the wild type Pfnek-2 and, therefore, has all the properties of an effective chemical genetic tool that could be employed to determine the cellular targets for Pfnek-2.

**Conclusions:**

Allelic replacement of wild-type Pfnek-2 with the mutated kinase will allow for targeted inhibition of Pfnek-2 with NCL-00016066 and hence pave the way for comparative studies aimed at understanding the biological role and transmission-blocking potential of Pfnek-2.

## Background

Despite the efficacy of artemisinin-based combination therapy (ACT) in the treatment of malaria, an estimated 214 million cases and 438,000 malaria deaths occurred globally in 2015 [1]. Together with alarming recent reports of the emergence of resistance to ACT [2–4], this highlights the urgent need for novel anti-malarial treatments.

Protein phosphorylation is known to play key roles in the progression of the *Plasmodium* life cycle through both the human host and the mosquito vector. Thirty-six of the approximately 90 kinases encoded in the parasite’s genome appear to play essential roles in key parasite processes, including invasion, proliferation and cyto-adherence [5], suggesting that targeting these protein kinases has therapeutic value [6]. Despite this, and the fact that protein kinases have proven successful targets in a number of human diseases, most notably cancer [7], no protein kinase inhibitors have yet reached the clinic for the treatment of malaria.

Amongst the protein kinases that play essential roles in the parasite’s life cycle are Pfnek-1 and Pfnek-2, members of the NIMA-related protein kinase family (which consists in total of four members Pfnek1-4) [8–12]. Whereas Pfnek-1 is considered essential for the completion of the erythrocytic asexual cycle [10, 12], Pfnek-2 is dispensable for asexual proliferation but essential for the completion of sexual development of the parasite in the mosquito vector [9]. It therefore represents a potential target for transmission-blocking drugs. Phylogenetic analysis of *Plasmodium* Neks [8] indicates that Pfnek-2 and Pfnek-4 form a cluster that is loosely associated with human Hsnek4.

In humans, there are 11 members of the Nek family with various roles in several processes. The involvement of human Hsnek2 in cell cycle regulation events, including centrosome disjunction, spindle assembly and the DNA damage response [13], has suggested that inhibitors to Hsnek2 might have clinical efficacy in the treatment of cancer [14–19]. There have been considerable efforts to generate inhibitors to Hsnek2 through the implementation of structure-based design to exploit unique structural features of the ATP binding pocket [20–23]. This has lead to the development of a compound, 4-(6-ethynyl-9H-purin-2-ylamino) benzene sulfonamide (NCL-00016066), that acts as an irreversible inhibitor of human Hsnek2. Structural studies have established that the inhibitory activity of NCL-00016066 results from the presence of the ethynyl group which forms an irreversible covalent link with cysteine-22 in the glycine rich loop [24].

Although phylogenetic studies have indicated that Hsnek2 and Pfnek-2 are not orthologues [8] they do however share several common features, including a large methionine gatekeeper residue, a bulky phenylalanine which restricts access for ATP-competitive protein kinase inhibitors and the conserved DaFG……..A/SPE sequence within the activation loop. One difference however is that parasite Pfneks lack the cysteine in the glycine rich loop with which NCL-00016066 forms a covalent linkage in Hsnek2. This position in Pfnek-2 (amino acid 24) is occupied by a valine. It can, therefore, be predicted that in the absence of a cysteine in position 24, NCL-00016066 would act as a very weak inhibitor of Pfnek-2 activity. Furthermore, by replacing valine 24 (Val24) in Pfnek-2 with a cysteine, a mutant kinase would be generated that would be sensitive to NCL-00016066 inhibition. The final prediction would be that in the cysteine mutant, NCL-00016066 would act in an irreversible manner by forming a covalent link with the substituted cysteine.

The data presented here show that these predictions are correct and that by substituting Val24 for cysteine in Pfnek-2, it is possible to generate a chemical genetic tool that could be used to dissect the in vivo function of Pfnek-2.

## Methods

### Materials

Unless otherwise stated all biochemicals and reagents were from Sigma-Aldrich.

### Parasite culture and treatment with NCL-00016066

*Plasmodium falciparum* blood stage 3D7 (wild type) parasites were grown (as previously described [25]) in complete RPMI 1640 medium (RPMI 1640 medium with 2 mM l-glutamine, 25 mM HEPES, 2 g/l NaHCO_3_, 27.2 mg/l hypoxanthine and 0.5% Albumax II, pH 7.4) using O^+^ human red blood cells at 37 °C in an incubator with 5% CO_2_, 5% O_2_ and 90% N_2_. Sorbitol treatment was used to synchronize the parasites [26]: parasites were treated with 5% sorbitol for 20 min at room temperature to lyse trophozoite and schizont stage parasites. Dead parasites were removed by two washes with incomplete RPMI medium (RPMI 1640 medium with 2 mM l-glutamine, 25 mM HEPES, pH 7.4). Following sorbitol treatment parasites were transferred to complete RPMI 1640 medium.

To determine the viability of parasites cultures that had reached the trophozoite stage, they were diluted to 0.3% parasitaemia and 2% haematocrit and treated with increasing concentrations of NCL-00016066 in a 96-well round bottom plate (Corning) and incubated for 72 h in an incubator with 5% CO_2_, 5% O_2_ and 90% N_2_. Several wells were treated with either 10% DMSO or 20 µM chloroquine as controls. They were then placed in a −80 °C freezer prior to lactate dehydrogenase (LDH) and SYBR Green I assays.

### SYBR Green I assay

Frozen plates were thawed for approximately 4 h at room temperature and 100 µl of SYBR Green I reaction buffer (20 mM Tris, 5 mM EDTA, pH 7.5, 0.08% Triton X-100, 0.008% saponin and 0.1 µl/ml SYBR Green I) was added to each well on the plate. The plate was shaken for 10 min to ensure mixing and incubated for 1 h at room temperature in the dark. The fluorescence was read using a CLARIOstar plate reader. (BMG Labtech GmbH, Germany).

### Lactate dehydrogenase (LDH) assay

Frozen plates were thawed for approximately 4 h at room temperature and 150 µl of LDH reaction buffer [100 mM Tris–HCl, pH 8.0, 150 mM sodium lactate, 150 µM APAD (3-Acetylpyridine adenine dinucleotide), 180 µM Nitrotetrazolium Blue Chloride, 0.7% Tween-20 and diaphorase (0.3 units/assay)] was added to each well on the plate. The plate was shaken for 10 min to ensure mixing and the absorbance at 595 nm was obtained using a CLARIOstar plate reader. (BMG Labtech GmBH, Germany).

### Pfnek-2 plasmid design

The synthetic full-length coding sequence for Pfnek-2 (PlasmoDB identifier *PF*3D7_0525900) was purchased (with codon optimization for *Escherichia coli* expression) from GeneArt™ Life Technologies. The codon optimized DNA sequence was as follows (the codon encoding V24 is highlighted in bold); ATGAGCAAACCGAAAATGATCGGTCCGTATGAAGTTGTGAAAAGCATTGGTCGTGGTAGCTTTGGTATT**GTT**ACCGCAGTTAAAGATGAAAATGAAAAAATCTTTGTGATTAAAGAACTGGATATTAGCTGCATGAATAACAAAGAAAAAATGAATGTGGTGAATGAAATTCGTGCCCTGATTAAAATGAGCGTGCATCCGTTTATTGTGCGCTATAAAGAAGCCTTTGTTGAAGATTGCGTTCTGTATGTTGCGATGGATTATTGCATTAATGGTGATCTGGGCAAAGTGATCAAAAAACACAAAGAACTGGAAACCCCGATTCCTGAGAAAAAAATCAAACGTTGGCTGCTGCAGATTATTATGGCCATCAAATTCATTCATGATAAAAAACTGATCCATCGTGATCTGAAATGCAATAACATCTTCCTGGATGAAAAAGAACGTGCCAAAATTGGTGATTTTGGTCTGGCCAAATTTATCGAACAGACAGAACAGACCAATACCCTGTGTGGCACCATTGGTTATATGGCACCGGAAATTTGCAAAAATATCAATTACAGCTTCCCTGCCGATATTTGGAGCCTGGGTATTATTCTGTATGAACTGATTAGCCTGAAACCGCCTTTTAAAAGCAATAACAGCAATATGCTGAGCGTGGCCCAGAAAATTTGTGAAGATGAACCGGATCCGCTGCCGGATAGCTTTAGCAAAGATCTGATTAATCTGTGCTATTGGATGCTGAAAAAAGATTGGAAAGATCGTCCGACCATCTACGATATTATCAGCACCGATTATATCCAGGATGAACTGCAGCTGTTTAAACGTGAAATGCTGCAAGAACGTAACAGCCAGATT.

The codon optimized sequence was cloned into a pET30a vector using *Nde*I and *Xho*I sites and a stop codon was added by site directed mutagenesis. The final construct used for protein expression contained an N-terminal His tag and a cleavable Tev sequence followed by the full length Pfnek-2 sequence.

### Cloning of Pfnek-2 and site-directed mutagenesis

The plasmid described above encoding wild type PfNek-2 was used as a template to generate Pfnek-2 V24C by site directed mutagenesis. The following primers were used forward (5′-gcattggtcgtggtagctttggtatttgtaccgcagttaaaga-3′) and reverse (5′-tctttaactgcggtacaaataccaaagctaccacgaccaatgc-3′).

### Bacterial protein expression

Full-length Pfnek-2 as a 6-HIS-tagged protein was expressed in *E. coli* BL21-Codon Plus(DE3)-RIPL strain (Agilent Technologies). Cells were treated with 1 mM IPTG when the optical density of the cell culture at 600 nm reached 0.6, and protein expression was induced overnight at 18 °C. For purification, cells were lysed in buffer [50 mM bicine, pH 8.0, 150 mM NaCl, 20 mM Imidazole, 1% glycerol, 1 mM DTT, Protease Inhibitor Cocktail (Roche)] [1 tablet per 10 ml (cOmplete™ ULTRA Tablets by Roche)] by sonication for 10 cycles of 10 s bursts at 10 mA with a 30 s rest between each cycle. During sonication the cells were kept on ice. Lysates were then centrifuged at 20,000*g* for 30 min. The resulting supernatants were loaded onto a 0.5 ml pre-equilibrated Ni–NTA Superflow resin (Qiagen) column, washed with buffer (50 mM bicine, pH 8.0, 150 mM NaCl, 20 mM Imidazole, 1% glycerol and 1 mM DTT) and proteins were eluted with the same buffer containing 400 mM Imidazole. Eluted proteins were dialysed against 50 mM bicine, pH 8.0, 150 mM NaCl, 1% glycerol and 1 mM DTT at 4 °C overnight. Dialysed Pfnek-2 protein was aliquoted and stored at −80 °C.

### In vitro Pfnek-2 kinase assay

Recombinant His-tagged WT or V24C mutant Pfnek-2 were assayed for protein kinase activity using myelin basic protein (MBP) as a substrate. 1.8 µg of either Pfnek-2 WT or Pfnek-2 V24C were pre-incubated at room temperature for 40 min with/without NCL-00016066 (1, 10 or 20 µM) in Pfnek-2 buffer (10 mM bicine, pH 8.0, 40 mM NaCl, 1% glycerol, 10 mM MgCl_2_, 4 mM imidazole, 1 mM DTT) in the presence of 5 µg MBP. The reaction was started by the addition of 5 µl 200 µM ATP/^32^P-ATP (0.0185 MBq per reaction)—total volume 20 µl. Following incubation of 30 min at 37 °C, the reactions were stopped by adding 10 µl 2× Laemmli buffer and proteins were separated by SDS–PAGE on 12% gels and stained with Coomassie blue. Dried gels were exposed to X-ray film.

### NCL-00016066 compound

The human Nek2 inhibitor, NCL-00016066 (gift from Professor Roger Griffin, University of Newcastle) was dissolved in DMSO to 10 mM and frozen in aliquots of 10 µl at −80 °C. These aliquots were kept in the dark and a fresh aliquot diluted prior to each kinase assay. All samples in each kinase assay were pre-incubated with NCL-00016066 for 40 min at room temperature and the reaction initiated by the addition of ATP/^32^P-ATP as previously described above.

### Immobilising Pfnek-2 (wild type) and Pfnek-2 (V24C) on Ni–NTA Superflow Beads—pre-incubation with inhibitor NCL-00016066

Recombinant Pfnek-2 wild type and Pfnek-2 V24C were purified as previously described and incubated (1:1) without/with 20 µM NCL-00016066 diluted in 10 mM bicine, pH 8.0, 40 mM NaCl, 10 mM MgCl_2_, 1% glycerol, 4 mM imidazole, 1 mM DTT at room temperature for 30 min. 50 µl Ni–NTA Superflow resin (Qiagen) was added and the samples made up to 500 µl in buffer (10 mM bicine, pH 8.0, 40 mM NaCl, 10 mM MgCl_2_, 1% glycerol, 4 mM imidazole, 1 mM DTT) and rotated for 1 h at room temperature. The beads were washed 3× in 500 µl buffer for 5 min rotation and supernatants discarded. The in vitro kinase assay protocol described above was followed and the enzyme immobilized on beads (washed and supernatant removed) is assumed to be 5 µl for the purpose of the kinase assay. During incubation at 37 °C the beads were gently re-suspended every 10 min.

### Preparation of samples for mass spectroscopy

Recombinant Pfnek-2 wild type and Pfnek-2 V24C were purified as previously described except glycerol was absent from all buffers. The absence of glycerol had no effect on the activity of the enzyme and was only added to previous preparations to stabilize the enzyme. Glycerol however interferes with the mass spectrometry and so was omitted here. Both wild type and mutant (V24C) Pfnek-2 were incubated (1:1) without/with 100 µM NCL-00016066 diluted in buffer (10 mM bicine, pH 8.0, 40 mM NaCl, 10 mM MgCl_2_, 4 mM imidazole, 1 mM DTT) at room temperature prior to mass spectrometry analysis.

### Mass spectrometry—identification of putative covalent bonding of NCL-00016066 to Pfnek-2

LC–MS was carried out using an RSLCnano HPLC system (Thermo Scientific) coupled to an LTQ-Orbitrap-Velos mass spectrometer (Thermo Scientific). Samples were loaded at 0.1 ml/min onto a Vydac C8 5 µm 250 mm × 1 mm I.D. reverse phase column (Grace Davison). The protein was desalted for 10 min in the loading buffer (0.1% formic acid) before elution using a 10 min linear gradient from 3 to 96% B (80% acetonitrile/0.1% formic acid). The output of the column was sprayed directly into the H-ESI2 electrospray ion source of the mass spectrometer maintained at 5 kV. The ion trap was set to acquire 10 microscans over the *m/z* range 800–1200 Da in positive ion mode. The maximum injection time for MS was 50 ms and the AGC target setting was 3e4. Protein charge-state distributions were viewed using the Xcalibur program (version 2.1.0.1139, Thermo Scientific) and molecular weight calculations made using ESIprot [27].

### Mass spectrometry analysis of Pfnek-2 autophosphorylation

Recombinant Pfnek-2 (4 µl of 13 mg/ml stock solution of enzyme) was incubated with/without 1 mM ATP in Pfnek-2 buffer (10 mM bicine, pH 8.0, 40 mM NaCl, 1% glycerol, 10 mM MgCl_2_, 4 mM imidazole, 1 mM DTT) for 30 min at 37 °C. The reaction was stopped by adding 10 µl 2× Laemmli buffer and proteins were separated by SDS–PAGE on a 12% gel. The dye front was removed and the gel placed in fixing solution [7% glacial acetic acid in 40% (v/v) methanol] for 1 h. The gel was stained with four parts colloidal blue to one part methanol and destained with 10% acetic acid in 25% (v/v). Protein bands were excised from the gel and cut into small pieces and washed three times with 200 μl of buffer B [20 ml 400 mM ammonium bicarbonate (3.16 g/100 ml) with 100% acetonitrile in ratio 1:1]. The gel pieces were then washed twice with 200 μl acetonitrile (100%) and gel pieces air dried.

The gel fragments were then incubated for 30 min at 60 °C in 100 µl 10 mM DTT in 50 mM TEAB buffer, pH 8.5. The liquid was removed and the fragments incubated in 100 µl 100 mM iodoacetamide in 50 mM TEAB buffer for 30 min at room temperature in the dark. The fragments were washed twice in buffer B for 20 min, washed briefly in acetonitrile (100%) followed by a further addition of acetonitrile for 10 min at room temperature followed by aspiration of the fluid and air drying. Trypsin solution (1 μg in 50 μl of 50 mM TEAB buffer per sample) was added and incubated at 37 °C overnight. The gel pieces were washed in 50 mM TEAB buffer for 5–10 min on a rotating platform. The digests and washes were combined and dried completely in a SpeedVac. The samples were then enriched for phosphopeptides as described below.

### IMAC enrichment of phosphopeptides for mass spectrometry

PHOS-Select iron affinity gel (Sigma) was equilibrated with 5 × 1 ml IMAC load/wash buffer [0.25 M acetic acid, 30% (v/v) acetonitrile]. 500 µl IMAC load/wash buffer was added to completely dry samples and 50 µl of 50% PHOS-Select slurry was added followed by rotating at room temperature for 1 h. Following incubation with IMAC beads, the samples were transferred onto Mobicol ‘Classic’ spin columns (2B Scientific Ltd), centrifuged for 30 s at 1000×*g*, and the flow-through (unbound peptides) collected and frozen. The IMAC beads were washed twice with 200 µl of IMAC load/wash buffer, once with 200 µl of HPLC grade water and eluted twice with 100 µl of solution containing 22 µl ammonia solution (Fisher Chemical, 35% stock), 300 µl acetonitrile to a total volume of 1 ml with HPLC grade water. Eluates were concentrated to a small volume (15–20 µl) in a Speedvac centrifuge and submitted for mass spectrometry analysis.

### Mass spectroscopy and data processing—identification of potential autophosphorylation sites

This was carried out as previously described [28] with one difference in that peptides were eluted from a reverse-phase PicoFrit capillary column (75 µm i.d. × 400 mm) over a period of 2 h.

## Results

### Characterization of Pfnek-2 and Pfnek-2 V24C mutant kinases

**Fig. 1:**
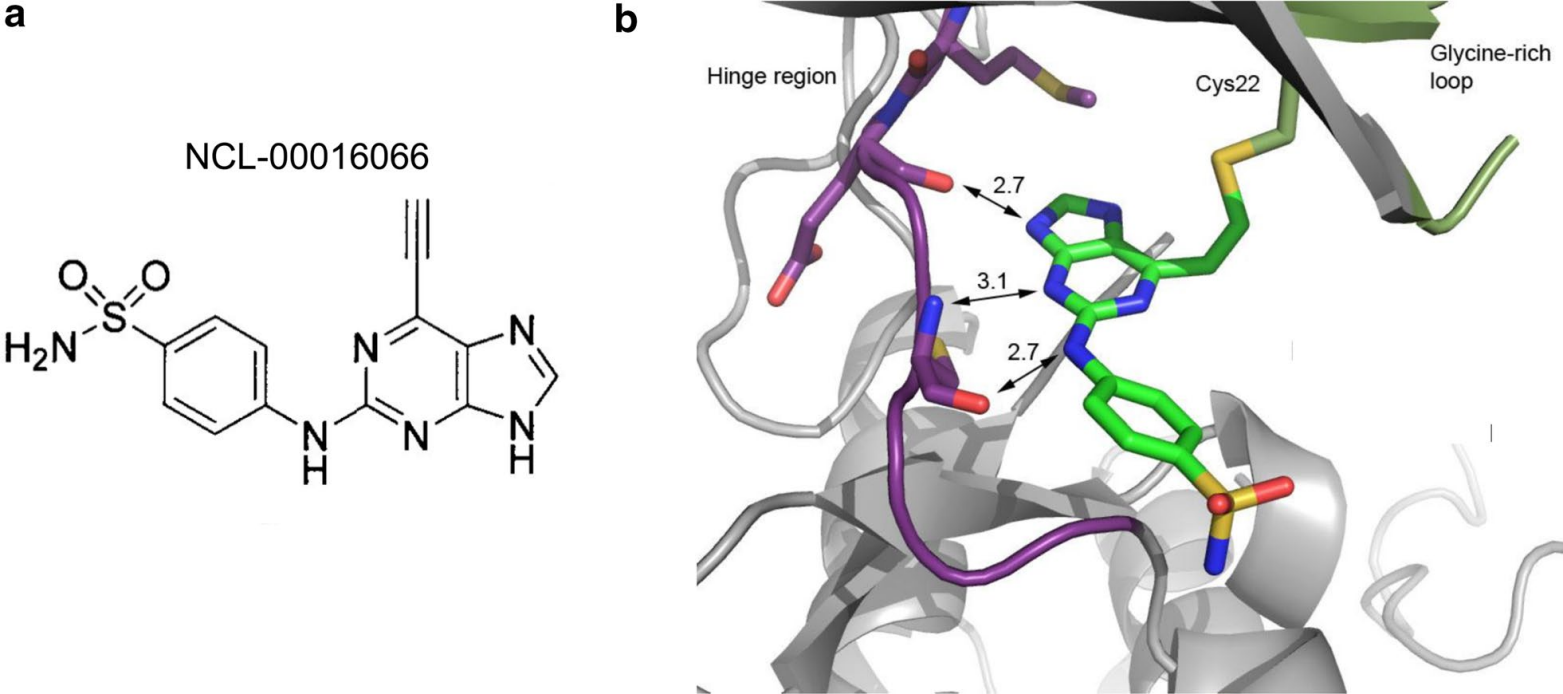
X-ray crystal structure of the ATP-binding domain of HsNek2 in complex with NCL-00016066 (determined at 2.0 Ǻ). **a** Chemical structure of NCL-00016066. **b** X-ray crystal structure of the ATP-binding domain (hinge region of kinase) of human nek2 in complex with NCL-00016066. Indicated is the presence of three predicted hydrogen bonds (*arrows*) between oxygens (*red*) and nitrogens (*blue*) within the hinge region and contained in NCL-00016066. The distances indicated are in angstroms. Also shown is the thioenol ether bond between cysteine 22 in Hsnek-2 and NCL-00016066 (*yellow*)

**Fig. 2:**
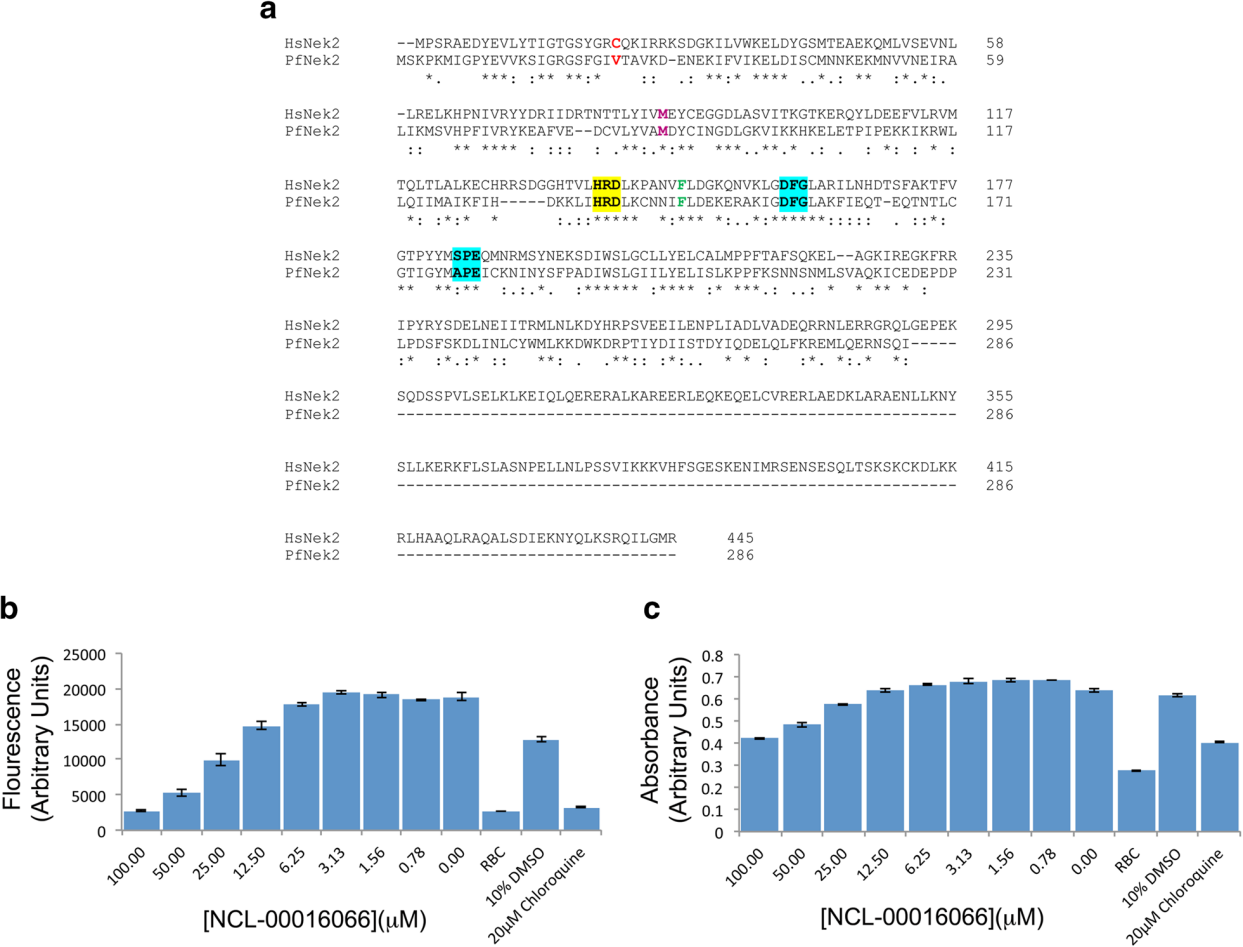
Alignment of HsNek2/Pfnek-2 protein sequences and the viability of parasite cultures following exposure to NCL-00016066. **a** Alignment of the amino acid sequences of Hsnek2 and Pfnek-2. The conserved methionine gatekeeper (*magenta*) and the conserved phenylalanine (*green*) are shown, as are the conserved HRD sequence (*yellow*) with the conserved activation loop motifs DFG and APE/SPE (*turquoise*). The valine at position 24 in Pfnek-2 is a cysteine in Hsnek2 and is highlighted in *red*. The identical amino acids between Hsnek-2 and Pfnek-2 are indicated with a *asterisk* (*), the highly conserved amino acids with *two dots* (:) and the conserved amino acids with *one dot* (.). **b**, **c** The viability of parasites in the presence of varying concentrations of NCL-00016066 as measured by (**b**) SYBR Green I and (**c**) LDH assays. As the parasites are chloroquine sensitive, chloroquine (20 μM) was included as a positive control while non-infected red blood cells, without inhibitor, and 10% DMSO were included as negative controls. The results are presented as the mean ± SEM. (n = 3)

**Fig. 3:**
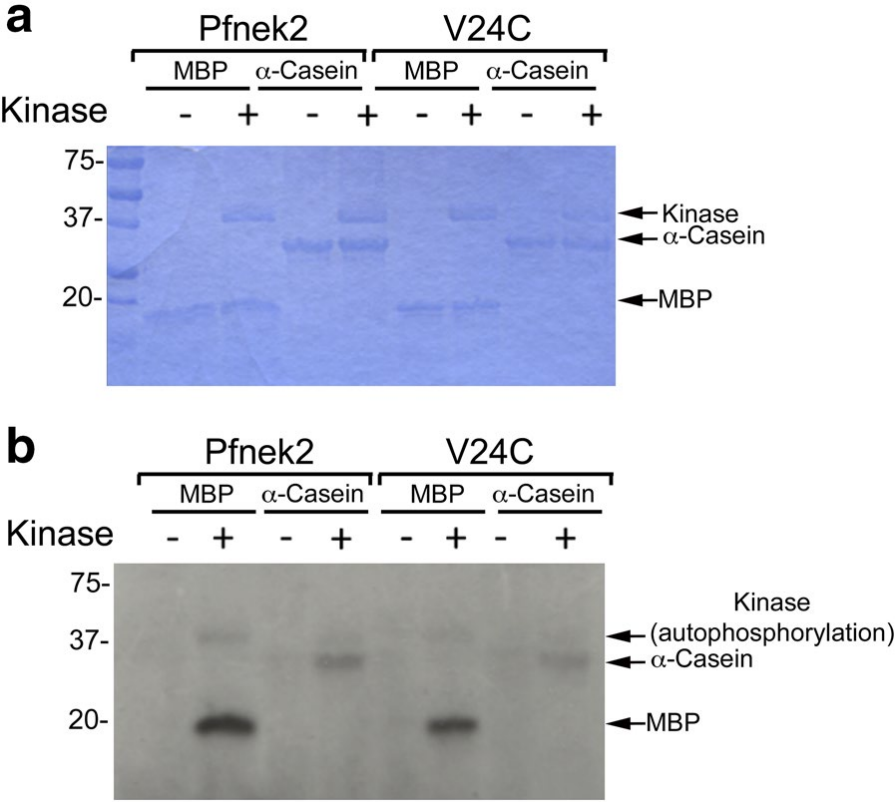
Phosphorylation of in vitro protein kinase substrates by Pfnek-2 and Pfnek-2(V24C). **a**, **b** Phosphorylation of the exogenous substrates myelin basic protein (MBP) and α-casein by Pfnek-2 and Pfnek-2(V24C). Shown is a Coomassie blue stained radioactive gel (**a**) and the resulting autoradiograph (**b**), which are representative of three experiments. The position of molecular weight markers (kDa) are shown

The compound NCL-00016066 (Fig. 1a) was originally developed as an inhibitor to human Hsnek2 [24]. A crystal structure of NCL-00016066 in complex with Hsnek2 was obtained, and confirmed that the inhibitor formed a covalent linkage with Cys22 in the glycine rich loop through the ethynyl moiety of NCL-00016066 (Fig. 1b). A crystal structure for Pfnek-2 is not currently available however amino acid sequence alignment of Hsnek2 with Pfnek-2 revealed that there are no cysteine residues within the glycine rich loop of Pfnek-2 (Fig. 2a); the equivalent position of Hsnek2 Cys22, in Pfnek-2, is occupied by Val24 (Fig. 2a). This would indicate that the parasiticidal activity of NCL-0016066 would be very low, which was indeed found to be the case with EC_50_ values of 23.95 ± 2.50 and 35.63 ± 3.03 µM as measured by SYBR Green I and LDH parasite death assays, respectively (Fig. 2b, c). Substituting Pfnek-2 Val24 with a cysteine created a mutant, Pfnek-2(V24C), that could be expressed in *E. coli* and affinity purified on a Ni–NTA chromatography column through a His-tag engineered into the N-terminus of the kinase. Purified Pfnek-2 and Pfnek-2(V24C) were resolved by SDS PAGE and appeared as a single band at approximately 38 kDa (Fig. 3a). Enzymatic activity of Pfnek-2 was assayed in in vitro kinase assays using α-casein or myelin basic protein (MBP) as exogenous substrate. Both wild type and mutant enzymes favoured MBP as a substrate (Fig. 3b) and appeared to display similar kinase activities. Subsequent experiments therefore used MBP as a substrate.

### Effect of NCL-00016066 on kinase activity

**Fig. 4:**
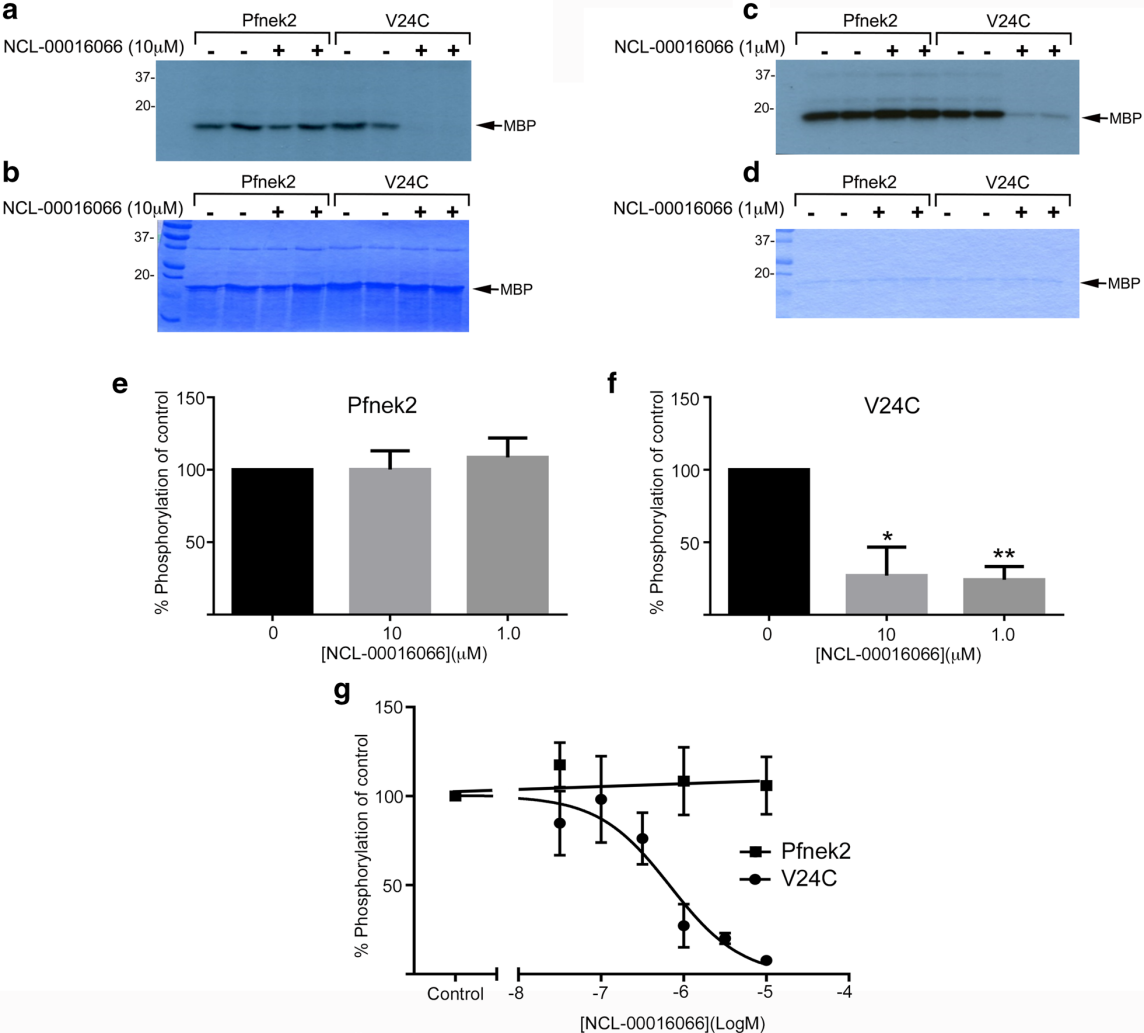
Inhibition of Pfnek-2 and Pfnek-2(V24C) kinase activity by NCL-00016066. **a**, **b** Representative experiment showing the effect of NCL-00016066 (10 μM) on the activity of Pfnek-2 and Pfnek-2(V24C). Shown is the autoradiograph (**a**) and Coomassie blue stained radioactive gel (**b**). **c**, **d** Same as (**a**, **b**) except the concentration of NCL-00016066 is 1 μM. The position of molecular weight markers (kDa) are shown. **e**, **f** Quantification of the experiments shown in (**a**–**d**). Data shown is the mean ± SEM; n = 3. Data analysed using Students paired *t* test *p ≤ 0.05. **g** Concentration–response curve for the activity of NCL-00016066 against Pfnek-2 and Pfnek-2(V24C). Data shown is the mean ± SEM; n = 3. Data analysed using students paired t-test *p ≤ 0.05

Having established the activity of the purified wild type and mutant kinases, we next tested the ability of NCL-00016066 to inhibit kinase activity. Purified kinases were incubated with MBP in the presence of 10 or 1 µM NCL-00016066 (Fig. 3a–g). In these experiments NCL-00016066 did not significantly affect the activity of wild-type Pfnek-2 (Fig. 4a–g). In contrast, NCL-00016066 acted in a dose-dependent manner to inhibit the activity of Pfnek-2(V24C), with an IC_50_ value of 501 nM [pIC_50_ (M) value, 6.30 ± 0.25 (n = 3)] (Fig. 4c, d, f, g).

**Fig. 5:**
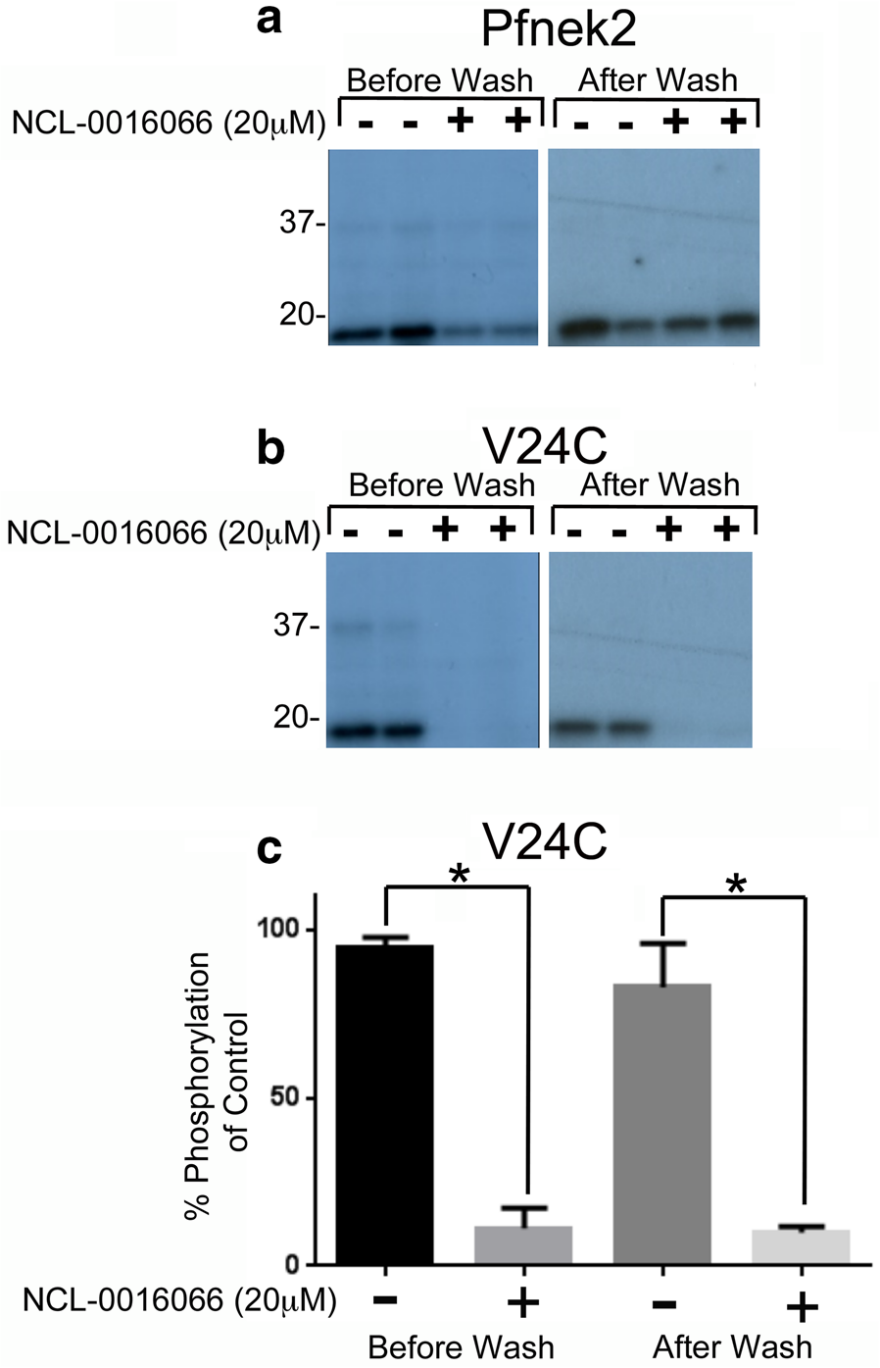
Irreversible inhibition of Pfnek-2(V24C) activity by NCL-00016066. **a**, **b** Activity of NCL-00016066 (20 μM) against Pfnek-2 (**a**) and Pfnek-2(V24C) (**b**) before and after wash out of the inhibitor. Shown are the autoradiographs (**a**, **b**). The position of molecular weight markers (kDa) are shown. **c** Quantification of three independent experiments indicating the effect of washing on Pfnek-2(V24C). Data shown is the mean ± S.E.M; n = 3. Data analysed using Students paired t-test *p ≤ 0.05

In order to investigate the mechanism of the inhibition of Pfnek-2(V24C) by NCL-00016066, the mutant kinase was pre-incubated with NCL-00016066 (20 µM) prior to immobilization on Ni–NTA beads, and unbound inhibitor were removed by extensive washing. As a control, Pfnek-2 was similarly incubated with high concentrations of NCL-00016066 (20 µM), which gave partial inhibition of kinase activity, which was reversed following washout (Fig. 5a). In contrast, NCL-00016066 (20 µM) completely inhibited the activity of Pfnek-2(V24C) (Fig. 5b, c) in a manner that was not reversed by the washout procedure (Fig. 5b, c). These data indicated that Pfnek-2(V24C) was sensitive to NCL-00016066 inhibition and that inhibition of the mutant kinase by NCL-00016066 was irreversible (Fig. 5b, c).

### Establishing the nature of the inhibition of the Pfnek-2(V24C) mutant

In an effort to further characterize the nature of the inhibition of the mutant Pfnek-2 with NCL-00016066, wild type Pfnek-2 or Pfnek-2(V24C) were pre-incubated with or without 100 µM NCL-00016066 and the intact protein subjected to analysis by mass spectrometry.

**Fig. 6:**
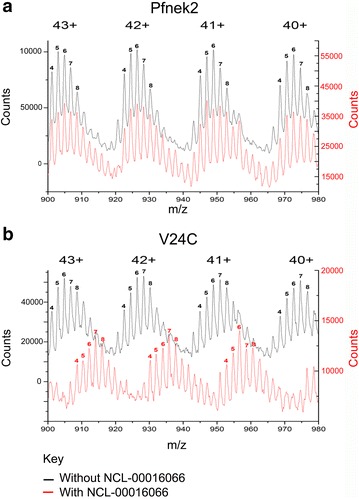
Shift in molecular weight of Pfnek-2 V24C as determined by mass spectroscopy. Pfnek-2 wild-type without (*black trace*) and with (*red trace*) NCL-00016066 **a** show no differences in peaks of the mass to charge ratios (*m/z*) for any particular charge state and number of phosphate groups (Table 1). In contrast when the mutant is incubated with inhibitor (**b**), there is a shift to the right (*red trace*) compared to without inhibitor (*black trace*) indicative of an increase in mass of 315.03 ± 0.53 Da—the approximate weight of the inhibitor confirming covalent bonding of the inhibitor to cysteine 22. For the purposes of clarity, in each panel the *red trace* is shifted vertically with respect to the *black trace*

Pfnek-2 without inhibitor (black trace Fig. 6a; Table 1) showed charge state distributions containing 4–8 peaks that corresponded to different phosphorylation states. The addition of NCL-00016066 did not change the *m/z* ratio of Pfnek-2 (red trace Fig. 6a; Table 1). In contrast, Pfnek-2(V24C) showed a clear shift in the *m/z* ratio upon addition of NCL-00016066 (Fig. 6b; Table 1). The shift corresponded to a molecular weight change of 315.03 ± 0.53 Da which is the mass of NCL-00016066 (Table 1). This strongly suggests a covalent linkage of NCL-00016066 with the mutant kinase.

**Table 1:** Molecular mass of Pfnek-2, Pfnek-2(V24C) and NCL-00016066

M+ #phospho	Charge state distribution							MW (Da)	Std Dev (Da)	MW (calc)	Δ (Da)
	44+	43+	42+	41+	40+	39+	38+				
Pfnek2+ vehicle											
5	882	903	924	947	971	995	1022	38,784	0.20	38,784	0.12
6	884	905	926	949	973	998	1024	38,864	0.72	38,864	0.31
7	886	907	928	951	975	1000	1026	38,944	0.68	38,944	0.30
8	888	909	930	953	977	1002	1028	39,024	0.51	39,024	0.42
Pfnek2+ NCL-00016066											
5	882	903	924	947	971	995	1022	38,784	0.80	38,784	0.02
6	884	905	926	949	973	998	1024	38,865	0.62	38,864	0.84
7	886	907	928	951	975	1000	1026	38,944	0.75	38,944	0.43
8	888	909	930	953	977	1002	1028	39,024	0.62	39,024	0.12
V24C+ vehicle											
5	883	903	924	947	971	995	1022	38,786	0.83	38,788	−1.58
6	884	905	926	949	973	998	1024	38,866	0.58	38,868	−1.71
7	886	907	928	951	975	1000	1026	38,946	1.27	38,948	−1.21
8	888	909	930	953	977	1002	1028	39,028	2.12	39,028	0.23
V24C+ NCL-00016066											
5	890	910	932	955	979	1004	1030	39,103	1.77	38,788	315.30
6	892	912	934	957	981	1006	1032	39,183	1.30	38,868	314.88
7	893	914	936	959	983	1008	1034	39,262	1.35	38,948	314.35
8	895	916	938	961	985	1010	1036	39,343	1.71	39,028	315.59

Predicted molecular weight (Predicted MW) = mass of kinase + phosphorylation sites

Δ (Da) = difference between predicted MW and measured MW for each charged state distribution

Table 1 shows the measured mass of the intact kinase [MW(Da)] and standard deviation, the predicted molecular weight for each charge state distributions (Predicted MW), the number of phosphate groups (M + Phospho), and the difference between the predicted and measured molecular weight [D(Da)]. The values highlighted in red indicate the difference in molecular weight of the mutant kinase in the presence of NCL-00016066 where the ∆(Da) value approximates to the molecular weight of the inhibitor.

### Autophosphorylation of Pfnek-2

**Fig. 7:**
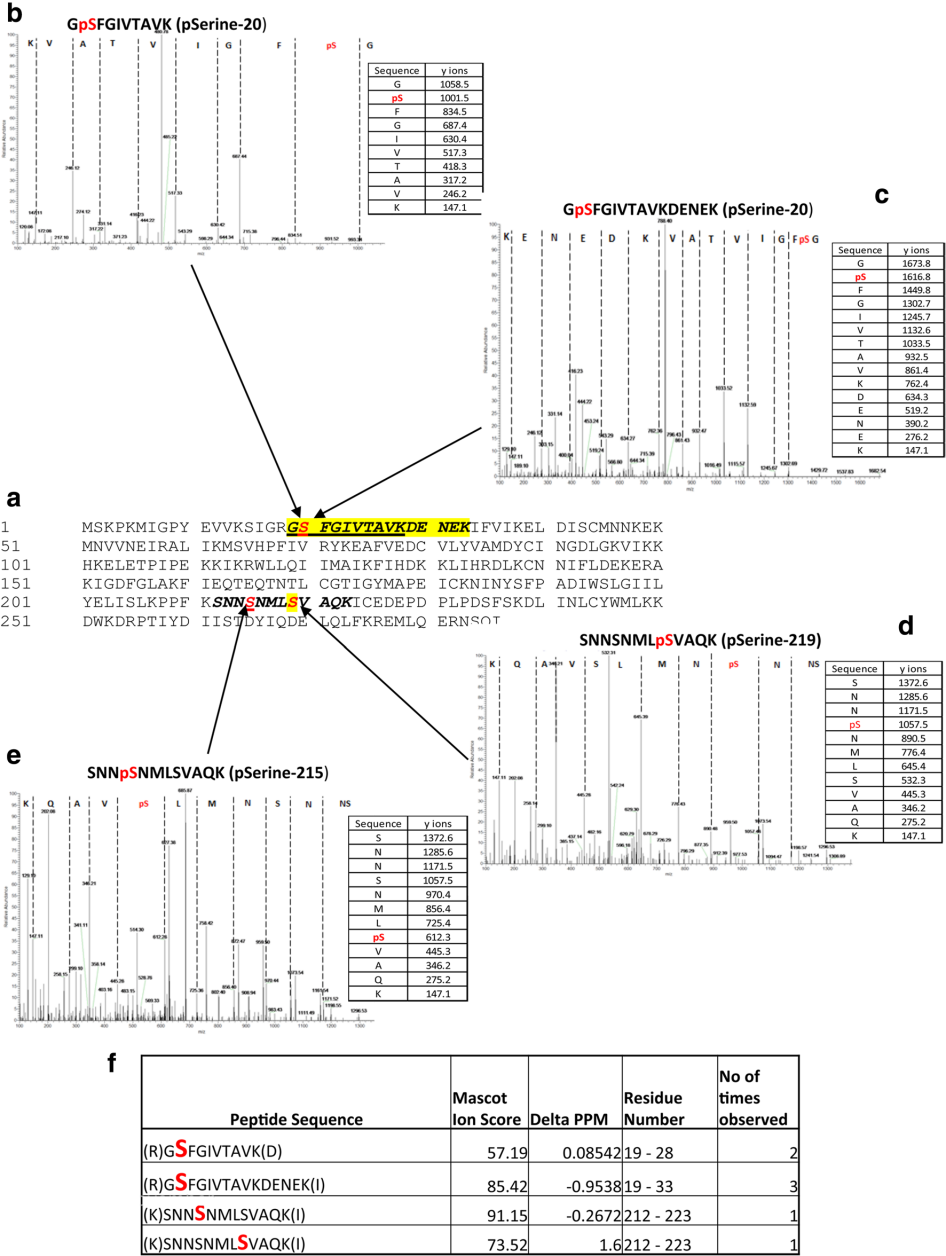
Autophosphorylation sites of Pfnek-2 as determined by mass spectroscopy. **a** Primary amino acid sequence of Pfnek-2.* Sections* in *bold* represent Pfnek-2 peptides observed in mass spectrometry experiments and in *red* are amino acids identified as being phosphorylated. **b**–**e** Representative mass spectra and associated fragmentation tables are shown that cover the three identified phosphorylated residues (Ser-20, Ser-215, and Ser-219). **f** List of the phospho-peptides identified in the overall data set

Mass spectrometry analysis of the intact protein as described above indicated that Pfnek-2 undergoes multiple autophosphorylation events. Analysis of tryptic phospho-peptides unequivocally identified three autophosphorylation sites, Ser20, Ser215 and Ser219 (Fig. 7). Interestingly, these autophosphorylation sites appeared to be distinct from those identified in Hsnek2 [29]. Only Ser20 is conserved in both Hsnek2 and Pfnek-2 but is not autophosphorylated in the human [29].

## Discussion

The aim of the current study was to engineer Pfnek2 in a manner that would confer sensitivity of the kinase to a kinase inhibitor that otherwise would show low levels of activity to wild type Pfnek2. This was achieved by mutating Val24 in the wild type Pfnek-2 to cysteine. This mutation confers susceptibility to the kinase inhibitor NCL-00016066, and that the nature of this inhibition is likely through an irreversible covalent link of NCL-00016066 with the modified cysteine.

Pfnek-2 is expressed predominantly in female gametocytes [10]. Transmission studies with the rodent malaria parasite *Plasmodium berghei* showed that parasites lacking the enzyme Pfnek-2 are able to undergo gametocytogenesis, gametogenesis and fertilization, but do not develop into ookinetes; this is likely due to a defect in meiosis, as *pbnek*-*2* ^−^ parasites appear to be unable to implement the pre-meiotic DNA replication that brings the DNA content to 4C in wild-type parasites, but remains 2C in the mutant parasites [10]. The demonstrated association of Pbnek-2 with microtubule-like structures in the gametocyte [10] suggests that the impairment in DNA replication may be mediated through improper spindle formation during the first round of meiotic DNA replication. Understanding the details of the molecular mechanisms that are regulated by Pfnek-2 (including identifying substrates of the enzyme) would be greatly facilitated by a model system whereby Pfnek-2 activity can be selectively and rapidly inhibited. The current study, has taken advantage of the extensive efforts to develop inhibitors to Hsnek2, considered a validated target in cancer drug discovery, to establish a chemical genetic approach to the study of Pfnek-2.

Structural analysis of the Hsnek2 in complex with NCL-00016066 demonstrated that this inhibitor acts in an irreversible manner by covalent linkage to cysteine 22 in the glycine rich loop. Interestingly, Pfnek-2 does not contain this cysteine, or any other cysteine, in this loop. Demonstrated here is the weak inhibitor activity of NCL-00016066 against wild-type Pfnek-2 likely due to a lack of a cysteine in this position. Consistent with this notion is that NCL-00016066 showed potent inhibitory activity if a cysteine residue is introduced into the glycine-rich loop of Pfnek-2. Furthermore, this inhibition appeared to be irreversible, and mass spectrometry studies indicated that this is likely due to covalent modification of the variant kinase at the substituted cysteine.

## Conclusions

Recent studies have deployed a chemical genetic approach to the study of PfPKG where parasites expressing a mutant form of the kinase that is resistant to inhibition by a selective PfPKG inhibitor was used to identify cellular targets and physiological roles of the kinase [28]. The engineering described here of a variant of Pfnek-2 that can impart sensitivity to chemical inhibition now allows for a similar chemical genetic strategy for the study of Pfnek-2. In this instance, the introduction of the Pfnek-2(V24C) mutant into the gene locus of Pfnek-2 will generate a mutant parasite strain that expresses a variant of Pfnek-2 sensitive to inhibition by NCL-00016066. In this way NCL-00016066 can be used as a selective probe to define the cellular targets and physiological role of this kinase. Importantly NCL-00016066 will likely be selective for the engineered mutant parasite as there are no cysteine residues in the glycine rich loop of any of the other Pfneks.

In this way, the data presented here opens the door to investigating the essential role that Pfnek-2 plays in the transmission of malaria from the host to the mosquito vector and in doing so might inform on the mode of action of potential transmission-blocking drugs targeting this kinase.

## Abbreviations

ACT: artemisinin-based combination therapy
MBP: myelin basic protein
Nek: NIMA-related kinase
NIMA: never in mitosis/*Aspergillus*
NTA: nitriloacetic acid
WHO: World Health Organization
